# COVID-19 and Sleep Disturbances: Unraveling the Connection

**DOI:** 10.3390/brainsci14030220

**Published:** 2024-02-27

**Authors:** Valentina Alfonsi, Serena Scarpelli, Maurizio Gorgoni

**Affiliations:** 1 Department of Psychology, Sapienza University of Rome, 00185 Rome, Italy; serena.scarpelli@uniroma1.it (S.S.); maurizio.gorgoni@uniroma1.it (M.G.); 2 IRCCS Fondazione Santa Lucia, 00179 Rome, Italy

## 1. Introduction

Since the beginning of the coronavirus (COVID-19) pandemic, a plethora of studies have been conducted to investigate the effects of this extraordinary phenomenon on sleep and mental health [1]. Research has consistently shown that the COVID-19 outbreak has been linked to several mental health disorders, particularly anxiety, depression, and stress-related disorders [2]. Furthermore, it is well known that sleep patterns underwent significant changes during the pandemic [3]. In particular, many studies described a decline in sleep quality and increased sleep disturbances, pre-sleep arousal, and sleep medication use. However, the impact of the pandemic on sleep appears to be highly variable, depending on the specific domain considered and several mediating factors [4]. Firstly, while sleep efficiency appears to be negatively influenced by forced confinement due to COVID-19, sleep duration and daytime functioning appear unchanged and—in some cases—have increased [5]. Secondly, the effects of the pandemic on sleep-related aspects vary among age ranges, clinical groups, and professional categories [6]. Furthermore, while some of the effects observed in the acute phase underwent spontaneous remission, other sleep–wake rhythms and dream activity changes were persistent even in subsequent pandemic waves [7]. 

To summarize, the relationship between sleep and COVID-19 is more complex than it seems. Studies investigating the specific role of crucial variables in determining the effects are necessary. This Special Issue aimed to collect insightful evidence to establish the role of clinical, socio-demographic, and environmental factors in modulating the impact of the pandemic on sleep, taking into consideration the different phases of the COVID-19 outbreak.

## 2. Special Issue Overview

For their article, Amicucci et al. (contribution 1) aimed to compare the impact of the COVID-19 lockdown on sleep health and psychological status between Italian adolescents and elderly people. They conducted a web-based survey study using validated questionnaires to assess sleep quality, insomnia symptoms, circadian typologies, depression, anxiety, and stress symptoms during the lockdown period for the first contagion wave of COVID-19 (25 March–3 May 2020) and they collected information about the perceived impact of the lockdown on sleep quality and habits. The statistical comparison highlighted a significant difference between the two groups. In particular, the young participants showed higher insomnia symptoms and a predominant evening chronotype compared to the elderly. Furthermore, the severity of depressive and stress symptoms was higher among adolescents then older people. Finally, young participants reported a more negative impact of the restraining measures on their sleep habits. For the first time, this study describes a higher level of resilience in older people in the face of pandemic-related stressful events in the Italian population. 

Cerasuolo et al. (in contribution 2) aim to clarify the discrepancies in the impact of the COVID-19 outbreak on children’s sleep quality by investigating possible age-related differences. They asked parents of toddlers (0–3 years) and preschoolers (4–5 years) to fill out an online survey on their children’s sleep patterns and habits, referring to both the lockdown period and the pre-pandemic period. Statistical comparisons between current and retrospective assessments of children’s sleep in the two groups show significant changes in sleep/wake schedules in the overall sample and improved sleep quality in preschoolers, while the scores for toddlers remain constant. 

Bruni and collaborators (contribution 3) employed an online retrospective survey to assess sleep patterns and disturbances in Italian children and adolescents with autism spectrum disorder (ASD) or attention deficit hyperactivity disorder (ADHD), compared to control children. They compared sleep-related aspects in all three groups, considering two consecutive periods (i.e., before and during the lockdown). The results confirm significant changes in several sleep features during the lockdown in all three groups and, interestingly, reveal the existence of specific changes in sleep habits consistent with the distinctive symptoms of the two neurodevelopmental disorders (e.g., greater sleep-schedule instability and increased weekday sleep-schedule delay in ADHD). 

The hospital-based cross-sectional survey conducted by Wańkowicz et al. (contribution 4) sought to examine the effect of the COVID-19 pandemic on sleep and mental health in people with a diagnosis of chronic disease. They considered two different groups as a function of the profession: workers in the healthcare sector (high-risk group) and workers in other occupations (low-risk group). Correlational analyses showed that the incidences of insomnia, anxiety, and depressive symptoms were more strongly associated with a pre-existent health condition than with profession (medical vs. non-medical). In particular, individuals with autoimmune diseases appeared to be the patients most at risk of a deterioration in their health condition during the pandemic.

Basishvili et al. (contribution 5) explore the persistence of the negative impact of the COVID-19 pandemic on sleep and mental health, focusing on the second-wave lockdown period in Georgia. Specifically, they used an online survey encompassing validated questionnaires to assess insomnia, pre-sleep arousal, psychosocial factors, and changes in sleep patterns in the general population. According to the hypothesis, the authors observed, even in the second lockdown period, the persistence of worse sleep quality, delayed bedtimes and risetimes, longer sleep latencies, higher awakenings, and shorter sleep durations than in the pre-pandemic period. Moreover, depression and COVID-19 infection were significantly predictive of an increased vulnerability to pre-sleep arousal, leading to a greater likelihood of developing insomnia disorder.

Gorgoni et al. (contribution 6) investigated the effects of the restrictive measures due to the COVID-19 pandemic through longitudinal evaluations conducted during different phases of the lockdown in Italy. They used an online survey to collect information about sleep, stress, and depressive symptoms and compare the conditions during and immediately after the lockdown. Their findings describe a decrease in stress levels and the persistence of sleep problems (i.e., poor sleep quality, clinically relevant pre-sleep arousal) and depressive symptoms after the end of the lockdown.

The article by Scarpelli et al. (contribution 7) sheds light on the evolution of dream activity during the second wave of COVID-19. The authors investigated sleep and dreaming aspects in the general population through an online survey containing several validated questionnaires. They compared the first and second waves of COVID-19, finding (a) lower dream-recall frequency, nightmare frequency, lucid-dream frequency, emotional intensity, and nightmare distress; and (b) a higher negative tone of dreams during the second wave of the pandemic than during the first. Furthermore, they observed significant differences concerning post-traumatic growth, sleep-related post-traumatic stress disorder (PTSD) symptoms, and sleep measures as a function of the changes in the oneiric frequency between the first and second waves. Finally, COVID-19-related factors (i.e., job changes, forced quarantine, having COVID-19-infected relatives/friends, or asking for mental health help) were confirmed as significant determinants of both qualitative and emotional dream features.

## 3. Conclusions

The collection of articles published in this Special Issue focuses on the effects of the COVID-19 outbreak on sleep and dream activity in different populations and over different time intervals. 

The purpose of this Special Issue was to observe the effects within specific samples grouped by age, clinical condition, or profession in order to highlight the different impacts of the pandemic on sleep and dream activity in relation to specific characteristics. Furthermore, the aforementioned studies investigated the temporal evolution of the observed effects, considering the different phases of the pandemic.

The ultimate goal is to emphasize the crucial aspects on which to base more tailored and effective treatment strategies.

All studies employed online surveys, which allowed for the collection of information from a broad sample of subjects even in the unique scenario of the pandemic and its related restrictions. However, we should consider that the use of online surveys entails a series of limitations. Firstly, the online recruitment strategy leads to a self-selection bias. Additionally, the survey represents a subjective tool that should be supported by the use of objective measures (e.g., actigraphy).

Overall, the observed results confirm that the abrupt changes in lifestyle conditions due to the spread of the COVID-19 virus have negatively affected sleep quality [8]. In particular, an increase in insomnia symptoms, greater pre-sleep arousal, and lower sleep efficiency have been observed compared to the pre-pandemic period, as confirmed by many other studies in the literature [9,10,11]. Furthermore, the rise in sleep and mental health problems has remained stable over time, underlining the persistence of these effects and the urgent need to adopt effective preventive and treatment strategies (contributions 5,6). Many studies have echoed and expanded upon these results, eventually describing the long COVID phenomenon [12].

Interestingly, the studies in this collection underline how the impact of the pandemic varies depending on specific protective or risk factors. According to previous studies [11], age plays a crucial role in mediating the effects of the pandemic on sleep-related aspects. For example, the comparison between adolescents and elderly people showed less resilience in the young population, who experienced more sleep problems following the pandemic (contribution 1). Furthermore, considering the comparison between toddlers and preschool children, we observed significant changes in sleep–wake patterns in preschoolers and unaltered conditions among toddlers (contribution 2). These results could indicate that age may represent a crucial factor, as different age groups exhibit various vulnerabilities to lifestyle changes. For example, adolescents and preschool children are more affected by environmental variations than elderly people and toddlers. Consequently, we can hypothesize that a greater degree of distress also has greater repercussions for both the quantitative and qualitative aspects of sleep [13].

Some articles in this Special Issue also focus on the role of pre-existing pathologies in modulating the effects of COVID-19 on sleep and mental health. On one hand, the results describe the congruence of the COVID-19-related alteration of sleep patterns with the typical symptoms of specific diagnoses (e.g., sleep fragmentation in children with ADHD) (contribution 3). On the other hand, a pre-existing condition appears to be a significant predictor of the onset of sleep problems during the pandemic, with particular reference to autoimmune diseases (contribution 4). Despite there being many studies describing the higher prevalence of sleep disturbances due to the COVID-19 pandemic in healthcare workers (i.e., at-risk professionals) [14], pre-existing chronic disease seems to play an even more important role than profession. 

Concerning the temporal evolution of pandemic effects on dream activity, a short-term effect was observed on the quantitative features of dreams and a persistent impact on the qualitative aspects, respectively, mirrored by a decrease in dream quantity and intensity over different pandemic waves and the maintaining of negative tones even at the end of the lockdown periods (contribution 7). 

In conclusion, although the negative consequences of the pandemic on sleep are widely documented [1,3], this Special Issue highlights relevant aspects that influence the outcomes [4] and that, consequently, should be kept in mind when planning prevention and treatment interventions. 
